# EEG Correlates of Ten Positive Emotions

**DOI:** 10.3389/fnhum.2017.00026

**Published:** 2017-01-26

**Authors:** Xin Hu, Jianwen Yu, Mengdi Song, Chun Yu, Fei Wang, Pei Sun, Daifa Wang, Dan Zhang

**Affiliations:** ^1^Department of Psychology, School of Social Sciences, Tsinghua UniversityBeijing, China; ^2^Department of Computer Science and Technology, Tsinghua UniversityBeijing, China; ^3^School of Biological Science and Medical Engineering, Beihang UniversityBeijing, China

**Keywords:** positive emotion, EEG, film clips, classification, spectral power

## Abstract

Compared with the well documented neurophysiological findings on negative emotions, much less is known about positive emotions. In the present study, we explored the EEG correlates of ten different positive emotions (joy, gratitude, serenity, interest, hope, pride, amusement, inspiration, awe, and love). A group of 20 participants were invited to watch 30 short film clips with their EEGs simultaneously recorded. Distinct topographical patterns for different positive emotions were found for the correlation coefficients between the subjective ratings on the ten positive emotions per film clip and the corresponding EEG spectral powers in different frequency bands. Based on the similarities of the participants’ ratings on the ten positive emotions, these emotions were further clustered into three representative clusters, as ‘encouragement’ for awe, gratitude, hope, inspiration, pride, ‘playfulness’ for amusement, joy, interest, and ‘harmony’ for love, serenity. Using the EEG spectral powers as features, both the binary classification on the higher and lower ratings on these positive emotions and the binary classification between the three positive emotion clusters, achieved accuracies of approximately 80% and above. To our knowledge, our study provides the first piece of evidence on the EEG correlates of different positive emotions.

## Introduction

Emotion is one of the most active and attractive fields in psychology. Compared to the extensive and detailed studies of negative emotions such as depression, fear, anger etc., positive emotions, however, are far less investigated. In recent years, the rise of positive psychology has inspired researchers toward the exploration of positive emotions (Fredrickson, 2013), aiming at a more balanced understanding of human emotions (Seligman, 2002; Seligman and Csikszentmihalyi, 2014).

Positive emotions have been suggested to be more than the counterpart of negative emotions in people’s daily life. In the seminal work by Fredrickson and Levenson (1998) and Fredrickson (2001), positive emotions are proposed to broaden people’s momentary thought-action repertoires, which over time serves to build their enduring personal skills and resources. Indeed, studies have shown that people’s momentary cognitive ability, such as the attentional scope, was broadened when primed with stimuli of positive emotions (Fredrickson and Branigan, 2005). People with a higher level of self-reported positive emotions are associated with enhanced ability to understand others’ emotions, which is a critical skill in building cooperation (Eisenberg and Miller, 1987; Zaki et al., 2008). Consequently, it has been reported that positive emotions are critical for adaptive social functioning, such as relationship fostering, commitment keeping, etc. (Berry and Hansen, 1996; Gonzaga et al., 2001; Gruber et al., 2011; Rand et al., 2015).

Despite the behavioral importance of positive emotions, their physiological mechanisms remain to be elucidated. Compared to negative emotions, positive emotions are generally believed to elicit less reactivity, if at all (Ekman et al., 1983; Cacioppo et al., 2000). For instance, it has been demonstrated that positive emotions did not elicit significant cardiovascular responses compared to a resting baseline, but they were capable of ‘undoing’ the physiological effects of negative emotions, allowing a faster recovery from negative emotions (Fredrickson and Levenson, 1998). In the meanwhile, there are some other studies reporting significant physiological responses to positive emotion stimuli, in both laboratory settings (Giuliani et al., 2008; Kreibig, 2010) and daily environments (Picard et al., 2001; Picard, 2010). Moreover, the findings on the brain regions responsible for processing positive emotions are much less consistent than those in negative emotions (Vytal and Hamann, 2010; Hamann, 2012). Only one type of positive emotion, i.e., happiness, has been reported in recent meta-analyses, with only reliable activations over rostral anterior cingulate cortex across studies (Vytal and Hamann, 2010). The absence of other positive emotions was partially explained by the limited number of studies on these subjects (Lench et al., 2011).

The lack of consistency in previous studies may be attributed to the oversimplified categorization of positive emotions, considering all positive emotions to have similar physiological responses. To date, physiological researches have either included one single positive emotion as the representative subject (‘happiness’ most frequently used but with varied definitions and manipulations), or treated ‘positive emotions’ as a homogeneous group. The possible differences within positive emotions, however, were largely neglected. In contrast, there are ample evidences indicating different positive emotions have different behavioral characteristics, from their triggers, processes to outcomes. For instance, one recent study reported that eight positive emotions (amusement, awe, contentment, gratitude, interest, joy, love, and pride) had distinct core relational themes and expressive displays (Campos et al., 2013). Hereby, it is necessary to have a more detailed categorization of positive emotions, and it is plausible to hypothesize that different positive emotions are operationalized by different underlying physiological mechanisms.

Recent efforts are beginning to provide evidence for distinct physiological responses in different positive emotional states. Shiota et al. (2011) investigated the autonomic nervous system (ANS) responses to five positive emotions (anticipatory enthusiasm, attachment love, nurturant love, amusement, and awe) elicited by pictures, and found distinguishable response patterns on multiple ANS measures associated with each individual emotion. This issue has also been addressed indirectly at the neurophysiological level using the EEG technique: emotion states with similar high valence levels but different (i.e., high vs. low) arousal levels elicited by video clips, can be recognized at well above chance level, using EEG signals (Koelstra et al., 2012; Soleymani et al., 2012; Wang et al., 2014; Chen et al., 2015). However, as the arousal-valence emotion model is not capable of adequately describing the variety of positive emotions (Fredrickson, 1998), a direct investigation of the neurophysiological responses for different positive emotions is necessary to further our understanding of human emotions.

In the present study, we conducted an exploratory investigation of the EEG correlates of different positive emotions. The participants watched a series of film clips with different positive emotions while their EEGs were recorded simultaneously. We followed the categorization of positive emotions from Fredrickson (2013), in which joy, gratitude, serenity, interest, hope, pride, amusement, inspiration, awe, and love were selected as the ten most representative positive emotions for their relatively high frequency of experience in people’s daily life. Audiovisual video clips were used for emotion elicitation, to maximize the emotional responses compared to auditory (e.g., music) or visual (e.g., pictures) stimuli alone. As suggested in previous EEG studies on emotion (Lin et al., 2010; Koelstra et al., 2012; Soleymani et al., 2012), we mainly focused on the spatial patterns of the EEG spectral power in different frequency bands during video watching. A univariate correlation analysis and a multivariate classification based method were adopted to establish the link between the subjective emotion ratings and the EEG activities.

## Materials and Methods

### Participants

Twenty college students (nine females, mean age 23 years, ranging 21 – 29 years) participated in the experiment as paid volunteers. All of them had normal hearing, normal or corrected-to-normal vision. Informed consent was obtained from all participants. The study was conducted in accordance with the Declaration of Helsinki and approved by the local Ethics Committee of Tsinghua University.

### Materials

Thirty film clips were used as the positive emotion stimuli in the present study. These film clips were selected in three steps. First, 103 initial stimuli were selected mainly from commercial films (at least 10 representative clips for each positive emotion, at least 30 s per clip) by a group of five frequent film viewers. Second, these stimuli were rated by five students independently, using 7-point Likert scales on the ten positive emotions. The stimuli with the top-three highest ratings for each positive emotion were selected as the first-round candidates, resulting in 30 stimuli. Third, five additional film clips were included based on the recommendations from the five independent raters. Another group of ten students was invited to rate these 35 stimuli and select the most representative 30 film clips. For each positive emotion, three clips with the highest ratings of the corresponding emotion were selected for the EEG experiment. In addition, one neutral stimulus and six negative emotion stimuli (three with high arousal and the other three with low arousal) were included as control. All the student raters are students from Department of Psychology, Tsinghua University, with good background knowledge on the ten positive emotions.

The film clips were edited to include a complete scene so that the participants could have a good understanding of the scenes and hereby be better engaged in the target emotions. The duration of the selected stimuli varied between 30 s to 129 s, with a mean value of 69 s and standard deviation of 24 s. The variability of the durations is comparable to existing film clip databases used in neurophysiological studies (e.g., Douglas-Cowie et al., 2007; Schaefer et al., 2010; Soleymani et al., 2012). For those film clips containing non-Chinese dialogs, Chinese subtitles were added to guarantee a full understanding of the contents. A complete list about the basic information of the selected film clips is provided in Supplementary Material.

### Procedure

The experiments were carried out in the laboratory environment with controlled illumination (at a normal office illumination level). The stimuli were displayed on an LCD monitor (22-inch, DELL, USA) with a 60 Hz refreshing rate. Stereo speakers (DELL, USA) were used and the sound volume was set at a fixed and comfortable level.

The participants watched all the 37 film clips (30 positive, 6 negative, and 1 neutral) with their EEG recorded. During the clip presentation, the participants were asked to immerse themselves in the film clips. After the completion of each clip presentation, the participants rated their emotional experiences on 7-point Likert scales on 14 items, including the ten positive emotions, as well as arousal, valence, familiarity and liking. The participants then took a rest for at least 45 s, and press the space button when he/she feels ready to watch the next video clip. They were asked to clear up their mind as best as they can during the rest period. Subjective ratings on all the emotional experience items were obtained for all stimuli, resulting in a multivariate description of each film clip. The negative and positive clips were presented separately rather than intermixedly, in order to avoid possible interactive influences between negative and positive clips.

Prior to the experiment, an introduction of the 14 emotional items and the experiment procedure were explained to the participants. Then, the participants performed two practice trials to get familiarized with the procedure, in which two positive emotion clips (not used in the formal experiment) were shown, each followed by the same emotional experience scale as that in the formal experiment. In the formal experiment, the participants were first presented with the neutral clip, following with the six negative emotion clips, and then with the 30 positive emotion clips. The orders of the clip presentations were randomized within the negative and positive emotion clips, respectively. The experiment lasted for approximately 2.5 h, including 0.5 h for EEG setup.

**FIGURE 1:**
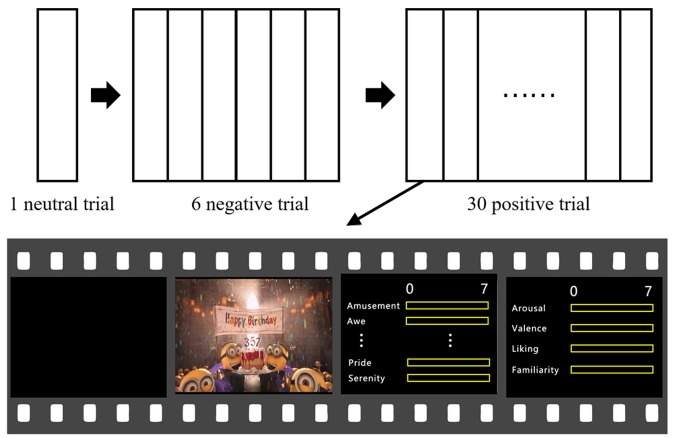
**Experiment procedure**.

The experiment procedure is shown in **Figure 1**. Presentation of the stimuli and the rating procedure were programmed in MATLAB (The Mathworks, USA) using the Psychophysics Toolbox 3.0 extensions (Brainard, 1997).

### EEG Recordings

EEGs were recorded from 32 electrodes (Fp1/2, Fz, F3/4, F7/8, FC1/2, FC5/6, Cz, C3/4, T3/4, CP1/2, CP5/6, Pz, P3/4, P7/8, PO3/4, PO7/8, Oz, O1/2), referenced to linked mastoids, with a forehead ground at Fz. A portable wireless EEG amplifier (NeuSen.W32, Neuracle, China) was used for data recording at a sampling rate of 250 Hz. Electrode impedances were kept below 10 kOhm for all electrodes.

### Data Analysis

#### Behavioral Data Analysis

To confirm whether the selected film clips elicited the expected positive emotions among the participants, the ratings on the ten positive emotion items were compared for each three film clips designated to elicit one specific positive emotion. Statistical analyses (repeated measures analysis of variance, rmANOVA) and *post hoc* paired *t*-tests were employed.

To obtain a general overview of the relationship between the ten positive emotions, pairwise correlation analyses were conducted on all pairs of cross-participant average ratings. As significant correlations were found among many pairs of positive emotions (see Results), we then applied unsupervised multidimensional scaling (MDS) method on the cross-participant average ratings per film clip, to further explore the similarity relationships across all the ten positive emotion experiences. These ten positive emotions were manually categorized into three clusters, on the basis of their geometric similarity in the MDS space. Subsequently, we also calculated the cluster scores for each film clip, by averaging the z-transformed ratings of all the emotions within each cluster, resulting in three cluster scores per film clip.

In addition, intra-class correlation coefficients (ICCs) were calculated for all the 14 emotional experience items, to explore the reliability of the ratings across participants.

#### EEG Preprocessing

The recorded EEG data were first notch filtered to remove the 50 Hz powerline noise, bandpass filtered to 0.05 – 50 Hz and then subjected to an artifact rejection procedure using independent component analysis (ICA). The independent components (ICs) with large weights over frontal or temporal areas, together with corresponding temporal course showing eye movement or muscle movement activities, were removed. The remaining ICs were then back-projected onto the scalp EEG channels, reconstructing the artifact-free EEG signals. About 1–2 ICs were rejected per participant.

#### Correlation Analysis

The EEG signals corresponding to the last 30 s of each film clip were extracted for further analysis, in order to obtain maximal emotional responses (following the procedure in Koelstra et al., 2012). The EEG signals from the 5-s time window before the presentation of the film clips were extracted as baseline. The spectral powers of each EEG channel were calculated using Fourier Transform and were then averaged separately over the frequency bands of theta (3 – 7 Hz), low alpha (8-10 Hz), alpha (8 – 13 Hz), beta (14 – 29 Hz) and gamma (30 – 47 Hz). The spectral powers in these five frequency bands during film clip watching were then baseline corrected by subtracting the corresponding spectral power during the baseline period. As all the film clips had ratings on all the emotional experience items, the response patterns to different positive emotion stimuli were characterized by computing the Pearson correlation between the spectral powers of each individual EEG channel and the subjective emotional ratings, for all the ten positive emotions as well as arousal, valence, familiarity and liking. The correlations were computed for each individual participant. For correlation calculations, EEG data from all film clips, including the negative and neutral clips, were used. The topographies of the across-participant average Pearson correlation coefficients between the rating of each emotional experience item and the EEG spectral power of each frequency band were expected to illustrate the neural responses to these positive emotions.

#### Classification Based Analysis

A classification based approach was then adopted to evaluate the overall contribution of all the EEG spectral powers to the representation of the ten positive emotions. A support vector machine (SVM) classification method was employed to perform a series of binary classifications for high and low rating values of each emotional experience. The features for classifications were the spectral powers over all the frequency bands from all the EEG channels, leading to 5 (frequency band) × 32 (channels) = 160 feature dimensions. These features were calculated on a basis of 1-s epochs from the 30-s clip-watching period, resulting in 900 samples from the positive emotion clips per participant. The segmentation into 1-s epochs was implemented to provide sufficient samples for more faithful classification accuracy estimation.

The 900 samples from the positive emotion clips were assigned into two classes based on the subjective ratings of their corresponding film clips with the median rating score as the threshold, leading to one class with the 450 samples with higher rating values on one emotional experience item and the other class including the 450 samples with lower rating values. Similar assignments were performed for the three MDS cluster scores as well; splitting the 900 samples into two classes of 450 samples each, with either larger cluster scores or smaller cluster scores. Taken together, 17 binary classifications (14 emotional experience ratings and 3 cluster scores) were carried out, to reveal the effectiveness of the EEG representation of the positive emotions.

The pairwise discriminability between different positive emotions were analyzed on the three extracted clusters, which gave a refined representation of the ten positive emotions. The pairwise analyses were not conducted on the original emotional experience items, as some of the original positive emotions showed high similarities in their ratings (as expressed by high pairwise correlations, see **Table 1** in Results). The same 1-s epoch based spectral power features were used. The labels for the 1-s epoch samples, were defined on the basis of the differences in cluster scores of their corresponding film clips. For example, film clips with higher score on cluster-1 than cluster-2 were considered to better represent cluster-1, while those with higher score on cluster-2 were better representations of the emotional content expressed by cluster-2. In this way, the numbers of film clips labeled as high and low score differences were 17/13, 14/16, and 15/15 for cluster-1 vs. cluster-2, cluster-1 vs. cluster-3, cluster-2 vs. cluster-3, respectively. Here cluster-1, cluster-2, and cluster-3 were the three defined MDS clusters (see Results for the emotions included in each cluster). By utilizing this labeling strategy, the pairwise binary classification based on the three representative clusters were expected to indicate the separability of different positive emotions on the EEG level.

**Table 1:** Pairwise correlation coefficients between the 14 emotional experience items.

	Amusement	Awe	Gratitude	Hope	Inspiration	Interest	Joy	Love	Pride	Serenity	Arousal	Valence	Familiarity	Liking
Amusement	–	-0.49^∗^	-0.10	0.13	0.25	0.75^∗^	0.85^∗^	0.17	0.15	-0.18	0.31^∗^	0.53 ^∗^	0.48^∗^	0.64^∗^
Awe		–	0.60^∗^	0.53^∗^	0.50^∗^	0.05	-0.17	0.15	0.54^∗^	0.48^∗^	-0.19	0.22	-0.06	0.15
Gratitude			–	0.91^∗^	0.82^∗^	0.36^∗^	0.33^∗^	0.75^∗^	0.81^∗^	0.51^∗^	-0.19	0.70^∗^	0.19	0.59^∗^
Hope				–	0.95^∗^	0.55^∗^	0.57^∗^	0.71^∗^	0.88^∗^	0.44^∗^	-0.07	0.86^∗^	0.34^∗^	0.75^∗^
Inspiration					–	0.67^∗^	0.65^∗^	0.56^∗^	0.93^∗^	0.30	0.12	0.88^∗^	0.48^∗^	0.81^∗^
Interest						–	0.88^∗^	0.38^∗^	0.56^∗^	0.17	0.21	0.80^∗^	0.54^∗^	0.89^∗^
Joy							–	0.47^∗^	0.56^∗^	0.11	0.21	0.85^∗^	0.60^∗^	0.90^∗^
Love								–	0.51^∗^	0.43^∗^	-0.24	0.66^∗^	0.09	0.58^∗^
Pride									–	0.28	0.14	0.80^∗^	0.54^∗^	0.75^∗^
Serenity										–	-0.78^∗^	0.36^∗^	0.03	0.30
Arousal											–	0.01	0.19	0.11
Valence												–	0.58^∗^	0.96^∗^
Familiarity													–	0.64^∗^
Liking														–

While the above mentioned classification procedures were performed for each individual participant, we also investigated the representation and separability of different positive emotions at the grand average level. To this end, similar labeling strategies were applied on the basis of grand average ratings and cluster scores. Accordingly, the EEG features were averaged across participants prior to classification.

All data analyses were conducted in MATLAB using the FieldTrip toolbox (Oostenveld et al., 2011). The SVM classifiers were implemented using LIBSVM (Chang and Lin, 2011) and all the reported results were based on 10-fold cross-validations.

## Results

The intra-class correlation coefficients (ICCs) of all the ratings on the 14 emotional experience items varied from 0.56 (serenity) to 0.95 (amusement), with a mean ICC of 0.87 (*SD* = 0.10), indicating good reliabilities across the participants.

**FIGURE 2:**
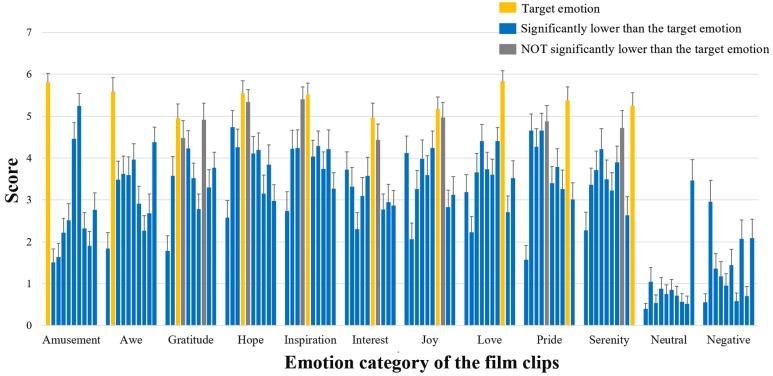
**Basic information of emotion ratings Participants’ ratings on the ten positive emotional experience items**. For each type of film clips, the ten bars indicate the mean ratings of these clips on amusement, awe, gratitude, hope, inspiration, interest, joy, love, pride, and serenity (from left to right). The bars in yellow show the ratings on the corresponding target emotion; The bars in blue suggest their ratings significantly lower than those of the target emotion (*post hoc* paired *t*-tests *p* < 0.05); The bars in gray indicate ratings NOT significantly lower than those of the target emotion (*p* > 0.05).

The film clips that expected to elicit one certain positive emotion indeed showed the highest ratings on the target emotion (rmANOVA *p* < 0.001 for all positive emotion items and *post hoc* paired *t*-tests showed significantly higher ratings for the target emotion in most cases, *p* < 0.05) (**Figure 2**). In most cases, the ratings on the target emotions were significantly higher than those on the remaining nine emotions, with only a few exceptions (e.g., ratings on ‘hope’ and ‘love’ did not differ from the ratings on ‘gratitude’, see the gray bars in **Figure 2**).

**FIGURE 3:**
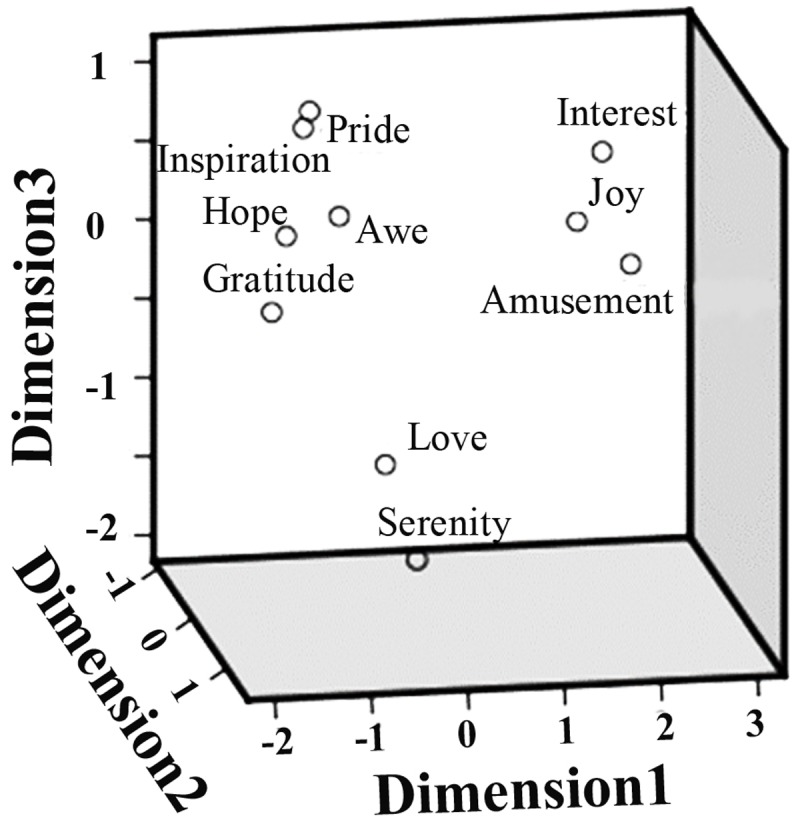
**Multidimensional scaling (MDS) space of ten positive emotions**. The MDS space showing the similarity of the ten positive emotions, based on the participants’ subjective ratings.

The pairwise correlation coefficients between the ratings on different positive emotions as well as arousal, valence, liking, and familiarity, are summarized in **Table 1**. Significant correlations were observed in many cases. For example, the participants’ ratings on ‘inspiration’ and ‘hope’ achieved a correlation coefficient of 0.95 (*p* < 0.05), indicating a considerable overlap between the feelings of inspiration and pride. The follow-up MDS analysis revealed a clear separation of these positive emotions in three clusters (**Figure 3**). A three dimensional MDS space was chosen, with the Kruskal’s Stress I value of 0.071. Cluster-1 is composed of awe, gratitude, hope, inspiration and pride, which is interpreted as ‘encouragement’; Cluster-2 is constituted by amusement, interest and joy, which is interpreted as ‘playfulness’; and Cluster-3 consists of love and serenity, which is interpreted as ‘harmony.’

**FIGURE 4:**
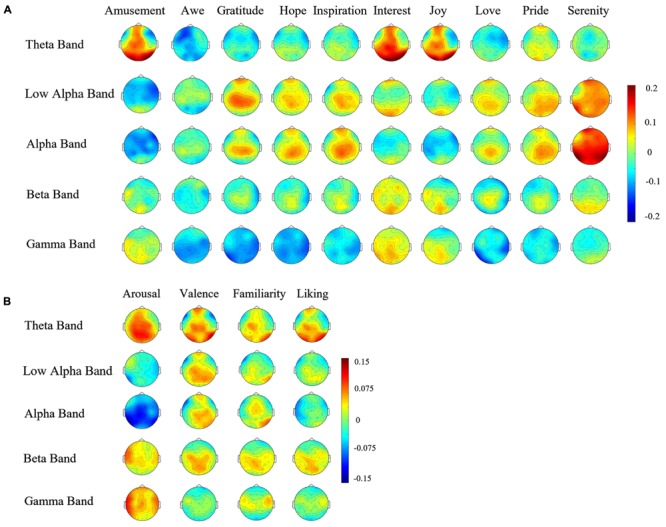
**(A)** Correlations between spectral power and ten positive emotion ratings Topographies of the correlation coefficients between the spectral powers in different frequency bands and the emotional experience ratings. **(B)** Correlations between spectral power and four emotion dimensions Topographies of the correlation coefficients between the spectral powers in different frequency bands and the emotional experience ratings on arousal, valence, familiarity, and liking.

**Figure 4A** shows the topographies of the average correlation coefficients between the spectral powers in the five frequency bands and participants’ ratings on the ten positive emotions. For the five ‘encouragement’ emotions (awe, gratitude, hope, inspiration, and pride), strong positive correlations were observed over the central area in both the alpha and the beta band. The three ‘playfulness’ emotions (amusement, interest, and joy) showed prominent positive correlations in the theta band power globally over most brain areas. The two ‘harmony’ emotions (love and serenity) showed strong correlations in the alpha band power over the parieto-occipital area. Different topographies were also observed in the gamma band for different emotions: a higher rating of the ‘playfulness’ emotions corresponded to an increase of gamma powers, while the other two clusters of emotions exhibited negative correlations. The topographies showing the correlations on arousal, valence, familiarity and liking are depicted in **Figure 4B**. The most notably patterns are the overall negative correlation between alpha power and arousal, and the frontal alpha asymmetry for valence.

**FIGURE 5:**
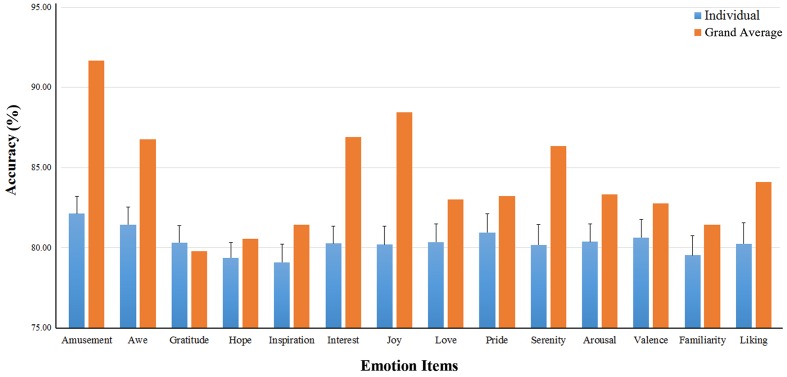
**Binary classification accuracies on the ten positive emotion experience**. The binary classification accuracies on the 14 emotional experience items. The blue bars show the individual-based accuracies and their standard deviations (the error bars); The yellow bars are the accuracies using the grand-average based features.

The binary classification accuracies on the 14 emotional experience items (for high vs. low ratings) are shown in **Table 2**. The mean accuracies on the ten positive emotions varied from 79.1 ± 5.1% (Inspiration) to 82.1 ± 4.9% (Amusement). The accuracies using the grand-average features were in general higher than the mean individual-based accuracies. On average, the grand-average based accuracies exceeded the individual-based accuracies by 3.9 ± 2.9%. Notably, amusement, joy, interest, serenity and awe showed a substantial increase over 5% (see **Table 2**; **Figure 5**).

**Table 2:** The binary classification accuracies on the 14 emotional experience items.

Participant	Accuracy (%)													
	Amusement	Awe	Gratitude	Hope	Inspiration	Interest	Joy	Love	Pride	Serenity	Arousal	Valence	Familiarity	Liking
1	74.2	84.1	76.6	84.8	86.3	82.0	76.1	79.7	83.4	77.4	81.3	80.4	74.7	78.7
2	84.0	81.7	84.6	83.2	76.6	78.9	85.2	83.7	84.9	85.7	87.0	84.2	76.6	76.0
3	81.1	83.0	85.1	80.9	83.6	80.1	86.4	82.8	82.0	84.4	82.3	82.9	79.8	83.2
4	77.9	73.9	74.3	70.6	68.8	72.4	67.3	76.1	76.1	70.6	73.1	68.9	69.4	73.1
5	83.6	78.8	82.3	78.9	84.3	82.9	78.1	80.2	84.8	82.8	79.9	83.1	83.3	86.3
6	79.8	79.2	83.7	82.4	74.7	75.2	78.1	73.3	78.4	86.6	87.3	82.7	78.9	82.4
7	85.7	82.4	85.8	82.7	82.8	83.7	83.4	82.3	80.4	82.0	81.0	85.4	86.3	79.9
8	76.4	73.8	72.9	73.9	79.0	79.2	77.9	76.4	82.6	79.7	77.7	80.6	85.3	77.6
9	86.6	89.2	80.2	78.4	81.3	80.0	83.6	80.4	91.8	83.2	78.2	81.4	81.8	87.1
10	76.6	79.7	74.7	75.3	77.3	80.8	76.1	76.8	75.2	73.9	74.1	74.6	72.2	76.2
11	78.2	77.4	77.9	75.7	75.2	76.3	75.8	75.7	71.2	72.7	75.8	72.1	76.8	74.3
12	83.2	80.1	80.0	81.0	72.2	83.2	85.1	77.2	81.3	74.4	74.6	75.2	72.2	72.7
13	82.4	80.8	84.2	81.3	81.2	75.0	76.8	88.3	79.8	74.3	75.8	82.0	81.6	82.0
14	87.0	86.0	82.7	80.0	78.4	82.6	86.6	79.1	79.4	83.8	82.3	82.1	81.3	82.8
15	88.6	89.0	83.8	80.8	78.6	88.2	82.4	83.9	81.7	79.7	82.9	87.7	81.8	87.6
16	77.8	72.4	69.9	72.2	72.4	69.7	75.2	71.8	72.9	72.3	75.3	76.6	73.9	67.9
17	90.6	88.9	88.0	87.9	88.2	87.4	86.6	93.0	89.9	90.6	90.0	84.4	92.0	92.2
18	76.2	81.9	81.3	77.1	75.8	78.6	75.8	80.2	75.9	80.1	77.2	78.0	79.4	80.9
19	83.2	83.8	78.8	80.4	84.2	85.4	82.4	84.0	84.3	81.9	83.0	82.1	79.8	80.9
20	89.6	82.8	79.7	79.7	80.9	83.6	84.8	82.0	82.8	87.4	88.3	88.1	83.6	83.1
Mean	82.1 ± 4.9	81.4 ± 4.8	80.3 ± 4.8	79.4 ± 4.3	79.1 ± 5.1	80.3 ± 4.8	80.2 ± 5.1	80.4 ± 5.0	80.9 ± 5.2	80.2 ± 5.7	80.4 ± 5.1	80.6 ± 5.0	79.5 ± 5.5	80.2 ± 5.9
Grand-Average	91.7	86.8	79.8	80.6	81.4	86.9	88.4	83.0	83.2	86.3	83.3	82.8	81.4	84.1

**FIGURE 6:**
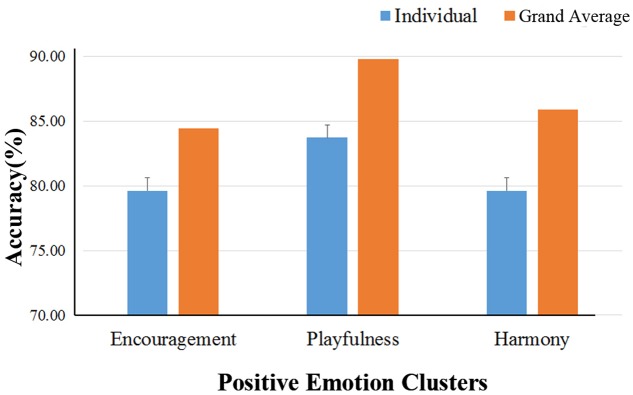
**Binary classification accuracies on the three positive emotion clusters**. The binary classification accuracies on the three positive emotion clusters. The blue bars show the individual-based accuracies and their standard deviations (the error bars); The yellow bars are the accuracies using the grand-average based features.

The binary classification accuracies on the three positive emotion clusters are shown in **Table 3**. The mean accuracies achieved 79.6 ± 4.9, 83.7 ± 4.6%, and 79.6 ± 5.3% for the emotion clusters of ‘encouragement,’ ‘playfulness’ and ‘harmony,’ respectively. The grand-average based accuracies were also higher, with an average increase of 4.9% (see **Table 3**; **Figure 6**). The pairwise classification accuracies between the three positive emotion clusters (higher vs. lower MDS cluster score differences) were listed in **Table 4**. The accuracies were 86.9% for ‘playfulness’ vs. ‘encouragement’, 85.2% for ‘encouragement’ vs. ‘harmony,’ and 84.3% for ‘playfulness’ vs. ‘harmony.’

**Table 3:** The binary classification accuracies on the three positive emotion clusters.

Participant	Accuracy(%)		
	Encouragement	Playfulness	Harmony
1	81.4	80.4	81.4
2	78.0	80.0	84.2
3	83.1	86.7	84.8
4	76.3	81.2	73.8
5	86.3	84.1	78.7
6	77.2	85.4	80.3
7	84.3	91.1	78.6
8	72.7	77.0	72.9
9	76.8	86.6	81.3
10	75.7	79.6	73.2
11	75.1	78.2	75.1
12	82.4	86.8	74.7
13	82.7	88.6	86.9
14	81.4	83.4	81.1
15	82.0	85.7	83.8
16	69.6	78.6	68.9
17	89.7	91.0	90.6
18	74.7	75.6	80.1
19	84.3	85.0	85.2
20	78.3	89.3	76.6
Mean	79.6 ± 4.9	83.7 ± 4.6	79.6 ± 5.3
Grand-Average	84.4	89.8	85.9

**Table 4:** The pairwise binary classification accuracies between the three positive emotion clusters.

	Accuracy (%)		
	Encouragement	Playfulness	Harmony
Encouragement	–	86.9	85.2
Playfulness		–	84.3
Harmony			–

## Discussion

In this study, we explored the EEG correlates of ten different positive emotions (joy, gratitude, serenity, interest, hope, pride, amusement, inspiration, awe, and love), using a video-watching paradigm. As shown by behavioral results, the selected film clips successfully elicited the target positive emotions. In addition, different positive emotions elicited different brain activation patterns, both spatially and spectrally. The positive emotions also showed a certain degree of similarity, resulting in three positive emotion clusters. Both the binary classification on the higher and lower ratings on these positive emotions and the binary classification between the three positive emotion clusters, achieved accuracies of approximately 80% and above.

First of all, the classical findings on emotions were replicated in the present study. For example, film clips with high arousal ratings showed a decreased parietal-occipital alpha power, compared to those with low arousal ratings, supporting the notion of alpha power as an effective index of the cortical arousal level (Coull, 1998; Barry et al., 2007). Film clips with high valence ratings, on the other hand, exhibited a right-lateralized frontal alpha activation, which is in line with the frontal alpha asymmetry theory (Davidson, 1984). As most previous studies have adopted the event-related paradigms, our film-watching design provides additional evidences from a more naturalistic perspective.

The effectiveness of the EEG correlates of these positive emotions was further explored by assessing their predictive power. The individual-based binary classification of higher or lower ratings on these positive emotions revealed well above chance-level accuracies, and these accuracies were comparable with those binary accuracies on arousal and valence in the present study. Moreover, our results on positive emotions are also comparable with, if not better than, the classification results on arousal and valence in previous studies: Classification accuracies between 52 and 78% have been reported in EEG-based studies using similar film watching paradigms (Koelstra et al., 2012; Soleymani et al., 2012; Wang et al., 2014; Chen et al., 2015).

Based on the similarity of the subjective ratings, those ten positive emotions were clustered into three main representative clusters, referred to as ‘encouragement,’ ‘playfulness’ and ‘harmony.’ The binary classification of higher or lower ratings on each of the emotion clusters, as well as their pairwise classification, suggested good discriminability as well. Especially, the pairwise classification results, together with the correlation topographies (**Figure 4**), suggest that the three positive emotion clusters might be associated with distinct underlying neural mechanisms at the EEG level.

Whereas the available literatures on the neural mechanisms of different positive emotions are limited, the topographies of the correlation coefficients between the spectral powers in different EEG frequency bands and the participants’ subjective emotional experience ratings may give us a clue on the EEG correlates of these positive emotions. The positive correlation between the alpha band powers over the frontal and central brain area and the ratings on the ‘encouragement’ emotions (awe, gratitude, hope, inspiration, pride) may be explained by the enhanced fronto-parietal alpha oscillation associated with cognitive processing (Palva and Palva, 2007), as these emotions requires more comprehension and cognitive process than the other positive emotions. Alternatively, the alpha activation can also be attributed to the mirror neuron system (MNS) (Oberman et al., 2007), as these emotions are more social-related than the other positive emotions. Indeed, alpha or mu rhythm has been reported previously to be connected with MNS (Muthukumaraswamy et al., 2004; Oberman et al., 2005; Pineda, 2005), although an opposite effect was observed in the present study possibly due to paradigm differences (event-related designs in most previous studies and a naturalistic film-watching design here). The global theta power changes that were shown to be correlated to the ‘playfulness’ emotions (amusement, interest, joy), are likely to reflect the participants’ fun-seeking, or approach motivation in general (Wacker and Gatt, 2010; Walden et al., 2015). While there are many other spatially or spectrally distinct patterns associated with different positive emotions (e.g., the beta and gamma correlation topographies, as well as detailed comparison of the theta and alpha results), more studies are necessary before providing further explanations on these findings.

One interesting finding in the present study is the consistently higher classification accuracies when using the grand-average EEG features, compared to the condition using the individual-based features. The result may seem surprising at a first glance, as human emotional experience is usually considered as very individualized. However, our results are in line with some recent studies proposing the inter-subject correlations of neural responses while perceiving the same emotional stimuli may be a good index of human emotion experiences, especially during conditions with complex and naturalistic stimuli (Dmochowski et al., 2012; Nummenmaa et al., 2012; Trost et al., 2015). Notably, all of the ‘playfulness’ emotions showed substantial increases of the accuracies, whereas the increases were less prominent for the other emotion clusters. The difference in the accuracy increase might be another indicator of the difference of the underlying neural mechanisms among the positive emotions in separate clusters: The film clips with high ‘playfulness’ emotional experiences may elicit more consistent neural responses among the participants. Further studies are necessary to fully address this issue.

Last but not least, there are several limitations of the present study that merit discussion. First, the selection of the ten positive emotions does not provide a complete coverage of all possible positive emotions, but rather expected to include the most representative ones. Second, the MDS-based clusters were based on the participants’ subjective ratings, and the ratings are constrained by the film clips in use. Hereby, the effectiveness of these clusters should be interpreted with cautious. Third, the film-watching paradigm has its own limitation in eliciting emotions. Emotion elicitation by other types of stimuli such as music, texts, or even self-generation, needs to be explored. In addition, the film clips in use are also limited, considering the degree of variation in clip features such as the duration, language, subtitles etc. Fourth, whereas the data analysis procedure in the present study mainly followed the most conventional approach using the classical spectral features, more advanced feature extraction and pattern recognition methods could be employed in future studies, with better classification performances expected (e.g., Chanel et al., 2009; Jin et al., 2014). Nevertheless, to our knowledge, our results provides the first piece of evidence on the EEG correlates of different positive emotions, and the binary classification accuracies based on 1-s epochs suggest that it is promising to implement brain-computer interfaces for real-time recognition of different positive emotions.

## Author Contributions

XH contributed to the conception design, data collection/analysis, and drafting the work; JY and MS contributed to the conception design and data collection; FW contributed to data analysis; PS and CY contributed to revising the work; DW contributed to the conception design, and drafting/revising the work; DZ contributed to the conception design, data interpretation, and drafting/revising the work. All authors approved the work for publication.

## Conflict of Interest Statement

The authors declare that the research was conducted in the absence of any commercial or financial relationships that could be construed as a potential conflict of interest.

## References

[B1] Barry R. J. Clarke A. R. Johnstone S. J. Magee C. A. Rushby J. A. (2007). EEG differences between eyes-closed and eyes-open resting conditions. *Clin. Neurophysiol.* 118 2765–2773. 10.1016/j.clinph.2007.07.028 17911042

[B2] Berry D. S. Hansen J. S. (1996). Positive affect, negative affect, and social interaction. *J. Personal. Soc. Psychol.* 71 796 10.1037/0022-3514.71.4.796

[B3] Brainard D. H. (1997). The psychophysics toolbox. *Spat. Vis.* 10 433–436. 10.1163/156856897X00357 9176952

[B4] Cacioppo J. T. Berntson G. G. Larsen J. T. Pohlmann K. M. Ito T. A. (2000). “The psychophysiology of emotion,” in *Handbook of Emotions*, 2nd Edn, eds Lewis M. Haviland-Jones J. M. (New York, NY: Guilford Press), 173–191.

[B5] Campos B. Shiota M. N. Keltner D. Gonzaga G. C. Goetz J. L. (2013). What is shared, what is different? Core relational themes and expressive displays of eight positive emotions. *Cogn. Emot.* 27 37–52. 10.1080/02699931.2012.683852 22716231

[B6] Chanel G. Kierkels J. J. Soleymani M. Pun T. (2009). Short-term emotion assessment in a recall paradigm. *Int. J. Hum. Comput. Stud.* 67 607–627. 10.1016/j.ijhcs.2009.03.005

[B7] Chang C. C. Lin C. J. (2011). LIBSVM: a library for support vector machines. *ACM Trans. Intell. Syst. Technol.* 2:27.

[B8] Chen M. Han J. Guo L. Wang J. Patras I. (2015). “Identifying valence and arousal levels via connectivity between EEG channels,” in *Proceedings of the 2015 International Conference on Affective Computing and Intelligent Interaction (ACII)*, (Rome: IEEE), 63–69.

[B9] Coull J. T. (1998). Neural correlates of attention and arousal: insights from electrophysiology, functional neuroimaging and psychopharmacology. *Prog. Neurobiol.* 55 343–361. 10.1016/S0301-0082(98)00011-2 9654384

[B10] Davidson R. J. (1984). “Affect, cognition and hemispheric specialization,” in *Emotion, Cognition and Behavior*, eds Izard C. E. Kagan J. Zajonc R. (New York, NY: Cambridge University Press), 320–365.

[B11] Dmochowski J. P. Sajda P. Dias J. Parra L. C. (2012). Correlated components of ongoing EEG point to emotionally laden attention–a possible marker of engagement? *Front. Hum. Neurosci.* 6:112 10.3389/fnhum.2012.00112 PMC3353265 22623915

[B12] Douglas-Cowie E. Cowie R. Sneddon I. Cox C. Lowry O. Mcrorie M. (2007). “The HUMAINE database: addressing the collection and annotation of naturalistic and induced emotional data,” in *International Conference on Affective Computing and Intelligent Interaction*, (Berlin: Springer), 488–500.

[B13] Eisenberg N. Miller P. A. (1987). The relation of empathy to prosocial and related behaviors. *Psychol. Bull.* 101:91 10.1037/0033-2909.101.1.91 3562705

[B14] Ekman P. Levenson R. W. Friesen W. V. (1983). Autonomic nervous system activity distinguishes among emotions. *Science* 221 1208–1210. 10.1126/science.6612338 6612338

[B15] Fredrickson B. L. (1998). What good are positive emotions? *Rev. Gen. Psychol.* 2 300–319. 10.1037/1089-2680.2.3.300 21850154 PMC3156001

[B16] Fredrickson B. L. (2001). The role of positive emotions in positive psychology: the broaden-and-build theory of positive emotions. *Am. Psychol.* 56:218 10.1037/0003-066X.56.3.218 PMC3122271 11315248

[B17] Fredrickson B. L. (2013). Positive emotions broaden and build. *Adv. Exp. Soc. Psychol.* 47 1–53. 10.1016/B978-0-12-407236-7.00001-2

[B18] Fredrickson B. L. Branigan C. (2005). Positive emotions broaden the scope of attention and thought-action repertoires. *Cogn. Emot.* 19 313–332. 10.1080/02699930441000238 21852891 PMC3156609

[B19] Fredrickson B. L. Levenson R. W. (1998). Positive emotions speed recovery from the cardiovascular sequelae of negative emotions. *Cogn. Emot.* 12 191–220. 10.1080/026999398379718 21852890 PMC3156608

[B20] Giuliani N. R. McRae K. Gross J. J. (2008). The up- and down- regulation of amusement: experiential, behavioral, and autonomic consequences. *Emotion* 8 714–719. 10.1037/a0013236 18837622 PMC4138973

[B21] Gonzaga G. C. Keltner D. Londahl E. A. Smith M. D. (2001). Love and the commitment problem in romantic relations and friendship. *J. Pers. Soc. Psychol.* 81:247 10.1037/0022-3514.81.2.247 11519930

[B22] Gruber J. Mauss I. B. Tamir M. (2011). A dark side of happiness? How, when, and why happiness is not always good. *Perspect. Psychol. Sci.* 6 222–233. 10.1177/1745691611406927 26168514

[B23] Hamann S. (2012). Mapping discrete and dimensional emotions onto the brain: controversies and consensus. *Trends Cogn. Sci.* 16 458–466. 10.1016/j.tics.2012.07.006 22890089

[B24] Jin J. Allison B. Z. Zhang Y. Wang X. Cichocki A. (2014). An ERP-based BCI using an oddball paradigm with different faces and reduced errors in critical functions. *Int. J. Neural Syst.* 24:1450027 10.1142/S0129065714500270 25182191

[B25] Koelstra S. Muhl C. Soleymani M. Lee J.-S. Yazdani A. Ebrahimi T. (2012). DEAP: a database for emotion analysis: using physiological signals. *IEEE Trans. Affect. Comput.* 3 18–31. 10.1109/T-AFFC.2011.15

[B26] Kreibig S. D. (2010). Autonomic nervous system activity in emotion: a review. *Biol. Psychol.* 84 394–421. 10.1016/j.biopsycho.2010.03.010 20371374

[B27] Lench H. C. Flores S. A. Bench S. W. (2011). Discrete emotions predict changes in cognition, judgment, experience, behavior, and physiology: a meta-analysis of experimental emotion elicitations. *Psychol. Bull.* 137 834–855. 10.1037/a0024244 21766999

[B28] Lin Y. P. Wang C. H. Jung T. P. Wu T. L. Jeng S. K. Duann J. R. (2010). EEG-based emotion recognition in music listening. *IEEE Trans. Biomed. Eng.* 57 1798–1806. 10.1109/TBME.2010.2048568 20442037

[B29] Muthukumaraswamy S. D. Johnson B. W. McNair N. A. (2004). Mu rhythm modulation during observation of an object-directed grasp. *Cogn. Brain Res.* 19 195–201. 10.1016/j.cogbrainres.2003.12.001 15019715

[B30] Nummenmaa L. Glerean E. Viinikainen M. Jääskeläinen I. P. Hari R. Sams M. (2012). Emotions promote social interaction by synchronizing brain activity across individuals. *Proc. Natl. Acad. Sci. U.S.A.* 109 9599–9604. 10.1073/pnas.1206095109 22623534 PMC3386135

[B31] Oberman L. M. Hubbard E. M. McCleery J. P. Altschuler E. L. Ramachandran V. S. Pineda J. A. (2005). EEG evidence for mirror neuron dysfunction in autism spectrum disorders. *Cogn. Brain Res.* 24 190–198. 10.1016/j.cogbrainres.2005.01.014 15993757

[B32] Oberman L. M. Pineda J. A. Ramachandran V. S. (2007). The human mirror neuron system: a link between action observation and social skills. *Soc. Cogn. Affect. Neurosci.* 2 62–66. 10.1093/scan/nsl022 18985120 PMC2555434

[B33] Oostenveld R. Fries P. Maris E. Schoffelen J. M. (2011). FieldTrip: open source software for advanced analysis of MEG, EEG, and invasive electrophysiological data. *Comput. Intell. Neurosci.* 2011:156869 10.1155/2011/156869 PMC3021840 21253357

[B34] Palva S. Palva J. M. (2007). New vistas for α-frequency band oscillations. *Trends Neurosci.* 30 150–158. 10.1016/j.tins.2007.02.001 17307258

[B35] Picard R. W. (2010). Emotion research by the people, for the people. *Emot. Rev.* 2 250–254. 10.1177/1754073910364256

[B36] Picard R. W. Vyzas E. Healey J. (2001). Toward machine emotional intelligence: analysis of affective physiological state. *IEEE Trans. Pattern Anal. Mach. Intell.* 23 1175–1191. 10.1109/34.954607

[B37] Pineda J. A. (2005). The functional significance of mu rhythms: translating ‘seeing’ and ‘hearing’ into ‘doing’. *Brain Res. Rev.* 50 57–68. 10.1016/j.brainresrev.2005.04.005 15925412

[B38] Rand D. G. Kraft-Todd G. Gruber J. (2015). The collective benefits of feeling good and letting go: positive emotion and (dis) inhibition interact to predict cooperative behavior. *PLoS ONE* 10:e0117426 10.1371/journal.pone.0117426 PMC4308081 25625722

[B39] Schaefer A. Nils F. Sanchez X. Philippot P. (2010). Assessing the effectiveness of a large database of emotion-eliciting films: a new tool for emotion researchers. *Cogn. Emot.* 24 1153–1172. 10.1080/02699930903274322

[B40] Seligman M. E. (2002). Positive psychology, positive prevention, and positive therapy. *Handb. Posit. Psychol.* 2 3–12.

[B41] Seligman M. E. Csikszentmihalyi M. (2014). *Positive Psychology: An Introduction*. Dordrecht: Springer, 279–298. 10.1037//0003-066x.55.1.5 11392865

[B42] Shiota M. N. Neufeld S. L. Yeung W. H. Moser S. E. Perea E. F. (2011). Feeling good: autonomic nervous system responding in five positive emotions. *Emotion* 11 1368–1378. 10.1037/a0024278 22142210

[B43] Soleymani M. Lichtenauer J. Pun T. Pantic M. (2012). A multimodal database for affect recognition and implicit tagging. *IEEE Trans. Affect. Comput.* 3 42–55. 10.1109/T-AFFC.2011.25

[B44] Trost W. Frühholz S. Cochrane T. Cojan Y. Vuilleumier P. (2015). Temporal dynamics of musical emotions examined through intersubject synchrony of brain activity. *Soc. Cogn. Affect. Neurosci.* 10 1705–1721. 10.1093/scan/nsv060 25994970 PMC4666110

[B45] Vytal K. Hamann S. (2010). Neuroimaging support for discrete neural correlates of basic emotions: a voxel-based meta-analysis. *J. Cogn. Neurosci.* 22 2864–2885. 10.1162/jocn.2009.21366 19929758

[B46] Wacker J. Gatt J. M. (2010). Resting posterior versus frontal delta/theta EEG activity is associated with extraversion and the COMT VAL 158 MET polymorphism. *Neurosci. Lett.* 478 88–92. 10.1016/j.neulet.2010.04.071 20450956

[B47] Walden K. Pornpattananangkul N. Curlee A. McAdams D. P. Nusslock R. (2015). Posterior versus frontal theta activity indexes approach motivation during affective autobiographical memories. *Cogn. Affect. Behav. Neurosci.* 15 132–144. 10.3758/s13415-014-0322-7 25245178 PMC5121190

[B48] Wang S. Zhu Y. Wu G. Ji Q. (2014). Hybrid video emotional tagging using users’. EEG and video content. *Multimed. Tools Appl.* 72 1257–1283. 10.1007/s11042-013-1450-8

[B49] Zaki J. Bolger N. Ochsner K. (2008). It takes two the interpersonal nature of empathic accuracy. *Psychol. Sci.* 19 399–404. 10.1111/j.1467-9280.2008.02099.x 18399894

