# Supplementary material for: EEG Correlates of Ten Positive Emotions
Source: Front Hum Neurosci. 2017 Jan 26;11:26. doi: 10.3389/fnhum.2017.00026 (PMC5266691; doi:10.3389/fnhum.2017.00026)
Supplement: Supplementary file 1 [file Table_1.DOCX]

Supplementary Material

**EEG Correlates of Ten Positive Emotions**

**Xin Hu^1^, Jianwen Yu^1^, Mengdi Song^1^, Chun Yu^2^, Fei Wang^1^, Pei Sun^1^, Daifang Wang^3^*, Dan Zhang^1^***

^1^ Department of Psychology, School of Social Sciences, Tsinghua University

^2^ Department of Computer Science and Technology, Tsinghua University

^3^ School of Biological Science and Medical Engineering, Beihang University

*** Correspondence:**

Dan Zhang, Room 334, Mingzhai Building, Tsinghua University, Haidian District, Beijing 100084, P.R. China, E-mail: [dzhang@tsinghua.edu.cn](mailto:dzhang@tsinghua.edu.cn)

Daifang Wang, E-mail: [daifa.wang@buaa.edu.cn](mailto:daifa.wang@buaa.edu.cn)

**Supplementary Table 1**

| **Clip Number** | **Film Name** | **Target Emotion** | **Start** | **End** | **Duration（s）** | **Language** |
| --- | --- | --- | --- | --- | --- | --- |
| 1 | My Neighbor Totoro | Joy | 0:05:12 | 0:05:46 | 34 | Japanese |
| 2 | Dallas Buyers Club | Gratitude | 1:26:44 | 1:28:24 | 100 | English |
| 3 | My Neighbor Totoro | Serenity | 0:35:10 | 0:35:44 | 34 | Japanese |
| 4 | Night at the Museum Ⅲ | interest | 0:10:05 | 0:10:42 | 37 | English |
| 5 | The Shawshank Redemption | Hope | 1:59:06 | 2:00:29 | 83 | English |
| 6 | Tsinghua Publicity Film | Pride | 0:00:33 | 0:01:50 | 77 | English |
| 7 | Forrest Gump | Inspiration | 1:55:33 | 1:57:42 | 129 | English |
| 8 | Modern Times | Amusement | 0:35:51 | 0:36:46 | 55 | English |
| 9 | Life of Pi | Awe | 1:11:56 | 1:13:25 | 89 | English |
| 10 | The Pursuit of Happiness | Love | 1:32:33 | 1:33:36 | 63 | English |
| 11 | Frozen | Joy | 0:04:12 | 0:05:01 | 49 | English |
| 12 | Les Choristes | Gratitude | 1:27:37 | 1:28:35 | 58 | French |
| 13 | The Tree of Life | Serenity | 0:39:00 | 0:39:51 | 51 | English |
| 14 | Inception | Interest | 0:29:46 | 0:30:53 | 67 | English |
| 15 | The Matrix Ⅲ | Serenity | 1:58:47 | 2:00:07 | 80 | English |
| 16 | Olympic Award Ceremony | Pride | 0:54:40 | 0:56:46 | 126 | English |
| 17 | The Theory of Everything | Inspiration | 1:51:09 | 1:52:26 | 77 | English |
| 18 | Minions | Amusement | 0:04:15 | 0:05:24 | 69 | English |
| 19 | The Tree of Life | Awe | 0:21:58 | 0:23:46 | 108 | English |
| 20 | Juno | Love | 1:22:17 | 1:23:02 | 83 | English |
| 21 | Ted | Joy | 0:02:01 | 0:02:46 | 45 | English |
| 22 | Schindler's List | Gratitude | 2:57:09 | 2:58:48 | 99 | English |
| 23 | Intouchables | Serenity | 1:42:11 | 1:43:18 | 67 | French |
| 24 | Harry Potter Ⅰ | Interest | 0:50:10 | 0:51:17 | 67 | English |
| 25 | Cast Away | Hope | 1:11:14 | 1:12:19 | 65 | English |
| 26 | Chinese National Day Parade | Pride | 0:59:41 | 1:00:26 | 45 | Chinese |
| 27 | The Pursuit of Happiness | Inspiration | 1:49:16 | 1:59:27 | 71 | English |
| 28 | Mr. Bean | Amusement | 0:08:40 | 0:09:53 | 73 | English |
| 29 | Baraka | Awe | 1:25:28 | 1:27:00 | 92 | English |
| 30 | Sex and the City Ⅱ | Love | 2:15:57 | 2:17:14 | 77 | English |
| 31 | Color Bars Video | Neutral | \ | \ | 30 | \ |
| 32 | The Pianist | High Arousal Negative | 0:56:50 | 0:57:59 | 69 | English |
| 33 | Hannibal | High Arousal Negative | 1:45:10 | 1:45:51 | 41 | English |
| 34 | The Shining | High Arousal Negative | 1:36:59 | 1:37:55 | 56 | English |
| 35 | Departures | Low Arousal Negative | 0:55:50 | 0:56:50 | 60 | Japanese |
| 36 | In Bruges | Low Arousal Negative | 0:24:47 | 0:25:32 | 45 | English |
| 37 | Gangs of New York | Low Arousal Negative | 2:34:55 | 2:36:16 | 81 | English |

Notes: the starting and ending time listed in the table will be different with different movie versions, so this information is for reference only.
